# Adoption barriers and facilitators of wearable health devices with AI integration: a patient-centred perspective

**DOI:** 10.3389/fmed.2025.1557054

**Published:** 2025-04-03

**Authors:** Haitham Alzghaibi

**Affiliations:** Department of Health Informatics, College of Applied Medical Sciences, Qassim University, Buraydah, Saudi Arabia

**Keywords:** wearable health devices, artificial intelligence (AI), chronic disease management, patient perceptions, digital health

## Abstract

**Introduction:**

Wearable devices that incorporate artificial intelligence (AI) have revolutionised healthcare through continuous monitoring, early detection, and tailored management of chronic diseases.

**Methods:**

This cross-sectional study analysed patients’ perceptions, trust, and awareness of AI-driven wearable health technologies, emphasising the identification of primary facilitators and barriers to adoption. A total of 455 participants, comprising individuals with chronic conditions, were recruited through convenience and stratified sampling methods. Data were collected via an online questionnaire that included demographic questions, Likert-scale items, and multiple-choice questions to evaluate awareness of particular AI features and the functionalities of wearable devices.

**Results and discussion:**

The findings indicated predominantly positive perceptions, with most participants concurring that wearable devices improve proactive care, facilitate remote consultations, and deliver precise health insights. Concerns regarding technical failures, data accuracy, and the potential reduction of human interaction were significant. No notable demographic differences were identified; however, participants with chronic conditions expressed more favourable perceptions. The research emphasises the necessity of user education, technical reliability, and professional oversight for the successful integration of AI-powered wearables in the management of chronic diseases.

## Introduction

In recent years, the amalgamation of wearable gadgets with artificial intelligence (AI) has transformed healthcare, enhancing patient care, diagnosis, and treatment (1–4). Wearable gadgets, including smartwatches, fitness trackers, and biosensor patches, provide continuous, real-time monitoring of essential health parameters such as heart rate, glucose levels, blood pressure, and sleep patterns (5–9). When integrated with AI, these devices can analyse extensive data sets to deliver actionable insights, improve clinical decision-making, and facilitate personalised, proactive healthcare (1, 10, 11).

### The advancement of wearable technology in healthcare

Wearable health technologies have advanced considerably, evolving from basic fitness trackers to complex medical-grade gadgets capable of continuous health monitoring. Recent breakthroughs in biosensor technology have facilitated the non-invasive monitoring of diverse biochemical and physiological signals (12). These advancements have established wearables as essential instruments in the management of chronic diseases, including diabetes, cardiovascular problems, and respiratory ailments (4, 13). Wearable devices provide continuous data streams, enabling patients and clinicians to monitor illness progression, identify early warning signals, and modify treatment programs as necessary (4, 14, 15).

The COVID-19 pandemic expedited the deployment of wearable health devices, enabling remote monitoring and bolstering telemedicine initiatives. Throughout this timeframe, technologies like smartwatches and biosensors were extensively utilised to monitor symptoms, assess healing, and diminish the necessity for in-person healthcare consultations. This underscored the essential function of wearables in preserving healthcare continuity during crises (15).

### The function of artificial intelligence in wearable healthcare technology

Although wearable devices produce significant health data, the sheer volume and intricacy of this information can be daunting for both patients and practitioners. AI tools are essential in this context. Machine learning and deep learning algorithms can scrutinize extensive datasets from wearables, uncovering trends and patterns that may not be readily observable (3, 10, 16, 17). AI-driven diagnostic models have exhibited significant precision in identifying illnesses such as atrial fibrillation, hypertension, and sleep disturbances. AI improves healthcare decision-making by analysing wearable data in real time, facilitating the early identification of potential health concerns and prompt actions (3).

AI-driven predictive analytics facilitates proactive care. For example, AI can evaluate continuous glucose monitoring data to forecast and avert diabetic crises or analyse electrocardiogram (ECG) signals from wearable devices to identify arrhythmias before to their manifestation (18, 19). This transition from reactive to proactive treatment allows healthcare providers to intervene early, enhancing patient outcomes and alleviating the strain on healthcare systems.

### Patients’ perception about wearable devices

Patient perspectives on wearable devices in healthcare underscore their potential advantages alongside prevailing concerns. Patients consider wearable devices essential for the ongoing monitoring of vital signs, physical activity, and sleep patterns, aiding in chronic disease prevention and health management. In a study, 55.8% of patients reported utilising a wearable device, whereas 95.3% of non-users expressed a willingness to adopt such technologies if offered at no cost (20).

Patients frequently acknowledge the advantages of wearable devices for tracking daily activities and monitoring health progress, which contribute to healthier lifestyles. Continuous monitoring is valued for its contribution to early detection and health maintenance. Nonetheless, privacy and data protection continue to be significant concerns for certain patients, who express apprehension regarding the possible misuse of their health data by various stakeholders (21, 22). Notably, over 50 % of patients in a particular study did not regard security risks as a significant issue (21).

Data sharing is largely regarded favourably, as evidenced by 98% of patients expressing a willingness to share wearable health data with researchers for health studies (21). Many patients have not yet shared device data with healthcare providers, yet they recognise its significance for enhancing healthcare outcomes (20).

Although perceptions are generally positive, obstacles to adoption persist. Cost is often identified as a major barrier, in addition to issues regarding the accuracy and reliability of wearable devices. Furthermore, certain patients, especially older adults, express a lack of familiarity with health self-monitoring (21). Socioeconomic factors significantly affect adoption rates; patients with higher incomes and those attending cardiovascular clinics demonstrate a greater likelihood of utilising wearable devices. Conversely, older age, male sex, and specific health conditions, including heart failure, correlate with reduced adoption rates.

### Study aim

To explore patients’ perceptions, awareness, and trust in wearable devices integrated with artificial intelligence (AI) for chronic disease management, identify key facilitators and barriers to adoption, and assess demographic variations influencing their attitudes towards AI-powered health technologies.

### Study objectives

- To assess patients’ overall perceptions and attitudes towards the use of wearable devices integrated with AI for chronic disease management.
- To evaluate patients’ levels of awareness and familiarity with key AI-driven features in wearable health devices, such as real-time alerts, predictive analytics, and virtual health assistance.
- To examine the extent of patient trust in AI-generated health insights and recommendations provided by wearable devices.
- To identify the main facilitators that enhance the adoption of AI-integrated wearable devices, such as personalised care and convenience in remote health monitoring.
- To identify key barriers to adoption, including concerns about data privacy, technical reliability, and the potential loss of human interaction in healthcare.

### What this study adds

- This study offers comprehensive insights into patients’ perceptions, trust, and concerns related to AI-integrated wearable devices, emphasising the behavioural factors that influence adoption.
- Comprehensive Awareness and Usage Assessment: This analysis enhances the understanding of patients’ awareness by evaluating their familiarity with particular AI-driven features and the functions of wearable devices.
- The study examines the impact of demographic factors, including age, gender, and chronic condition status, on perceptions of wearable health technologies.
- Examination of AI Tools for Chronic Disease Management: This study assesses participants’ preparedness to embrace AI-driven features, including virtual assistants and real-time notifications, thereby advancing research on AI’s contribution to proactive healthcare.
- The study identifies significant barriers to adoption, such as concerns regarding technical failures and data accuracy, while emphasising the necessity for professional supervision. These findings provide actionable insights for enhancing adoption strategies.

## Methods

This cross-sectional study assessed patients’ perceptions of wearable devices that incorporate artificial intelligence (AI) for healthcare applications, with a focus on chronic disease management. The cross-sectional approach offered a thorough evaluation of participants’ views on the advantages and obstacles associated with the adoption of wearable health technologies at a specific moment.

### Demographics and sampling

The research focused on patients possessing diverse levels of experience with wearable health devices, encompassing individuals with chronic conditions. A convenience sampling method was utilised for participant recruitment because of its efficiency. The sample size included 455 patients, providing a diverse representation across age, gender, and health status. Stratified sampling was employed to ensure proportional representation of patients with varying chronic conditions, frequencies of wearable device usage, and degrees of familiarity with wearable technology. Participants were recruited via online outreach, clinic announcements, and patient groups.

The process of data collection involves systematic gathering of information for analysis. It encompasses various methodologies to ensure accuracy and reliability of the data obtained.

The data collection period lasted 2 months, beginning in October 2024. An online questionnaire was distributed via a secure survey platform (Google forms), and participants received a direct link to the survey. Two reminder messages were dispatched during the third and sixth weeks of data collection to enhance participation. The online data collection method enabled access to a diverse array of participants, thereby ensuring a representative sample.

### Instrument for data collection

This study utilised an online questionnaire as the primary data collection tool to assess participants’ perceptions of wearable devices integrated with artificial intelligence (AI) in healthcare, emphasising potential benefits, challenges, and barriers to adoption. The questionnaire comprised five primary sections. The initial section presented a letter of assurance detailing the study’s purpose and nature, ethical considerations, and participants’ rights, with a focus on voluntary participation, confidentiality, and data anonymisation. The second section gathered demographic information, encompassing age group, gender, academic department, academic role or level, chronic condition status, familiarity with wearable health devices, and frequency of usage. This facilitated a thorough representation of participants from various demographic categories. The third section examined participants’ perceptions of AI-integrated wearable devices, consisting of 18 Likert-scale items that evaluated opinions on personalised care, trust in data accuracy, patient engagement, concerns regarding human interaction, and technical reliability. Responses were measured using a five-point Likert scale, with options ranging from “Strongly Disagree” (1) to “Strongly Agree” (5).

The fourth section included four multiple-choice questions aimed at evaluating participants’ awareness of AI features for chronic disease management, essential characteristics of wearable devices, types of wearable devices recognised by participants, and the intended uses of these devices. The questions enabled participants to choose multiple options, offering insights into their familiarity with and preferences regarding specific AI-driven functionalities, wearable device capabilities, and health monitoring objectives. The final section assessed participants’ perspectives on AI-driven tools, including virtual health assistants and chatbots, regarding their capacity to alleviate physician workload, deliver real-time insights, facilitate remote consultations, and encourage proactive healthcare.

The questionnaire was developed based on pertinent literature and validated via a pilot study with 8 patients to ensure clarity, readability, and relevance. Minor modifications were implemented in response to feedback to improve the construct validity of the instrument. The final questionnaire was developed to systematically capture participants’ experiences and perceptions, identifying patterns of agreement, concerns, and demographic variations associated with AI-integrated wearable health technologies.

### Data analysis procedure

Data analysis utilised SPSS (version 29) and R (version 4.3.0). Descriptive statistics, including frequencies, percentages, and mean scores, were calculated to summarise participant responses. Cronbach’s alpha was employed to assess the reliability of the Likert-scale items.

The Kruskal-Wallis and Mann–Whitney U tests were employed to evaluate differences in perceptions related to demographic and belief-based variables. The analysis indicated no statistically significant differences in perceptions related to familiarity with wearable devices (*p* = 0.87), frequency of device usage (*p* = 0.59), chronic condition status (*p* = 0.35), or gender (*p* = 0.65). The findings suggest that perceptions were largely uniform among the groups.

## Results

**Figure 1:**
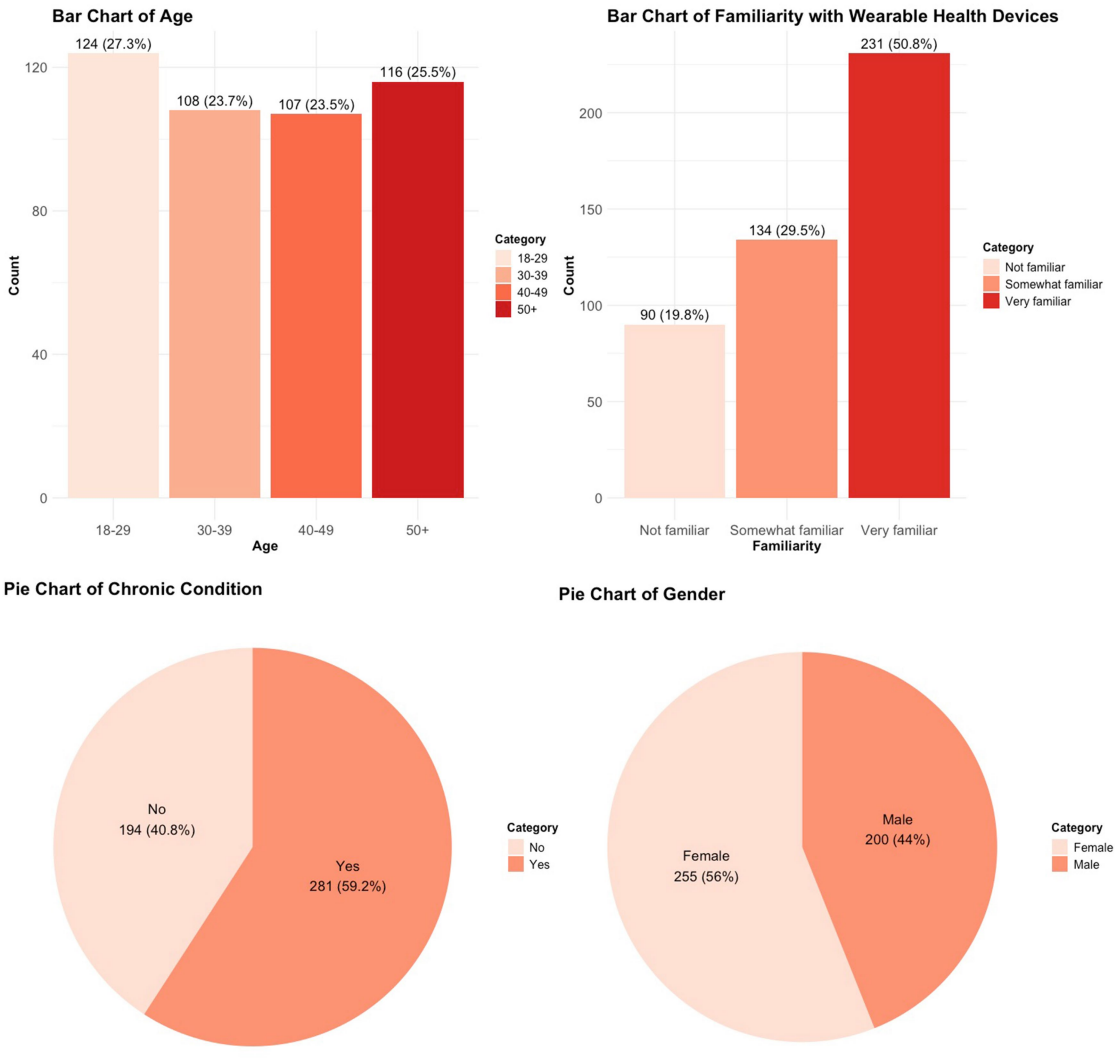
Demographics of the patients.

The bar charts in Figure 1 illustrate the demographic distribution of participants, offering insights into age, gender, chronic condition status, and familiarity with wearable health devices. The age distribution indicates that the predominant group of respondents (124) was aged 18–29, with nearly equal representation in the 30–39 (108), 40–49 (107), and 50+ (116) age categories. Diverse representation guarantees a range of perspectives on wearable health devices.

The gender distribution reveals a slight majority of female respondents (255) over male respondents (200), indicating a representative sample for evaluating gender-related perceptions. The bar chart depicting chronic condition status indicates that a majority of participants (261) reported the presence of at least one chronic condition, highlighting a significant population for assessing the application of wearable devices in chronic disease management.

The majority of respondents (231) reported being “somewhat familiar” with wearable health devices, followed by those who were “very familiar” (134) and “not familiar” (90). This distribution illustrates differing levels of awareness and experience with wearable technologies, potentially affecting perceptions and readiness for adoption.

**Figure 2:**
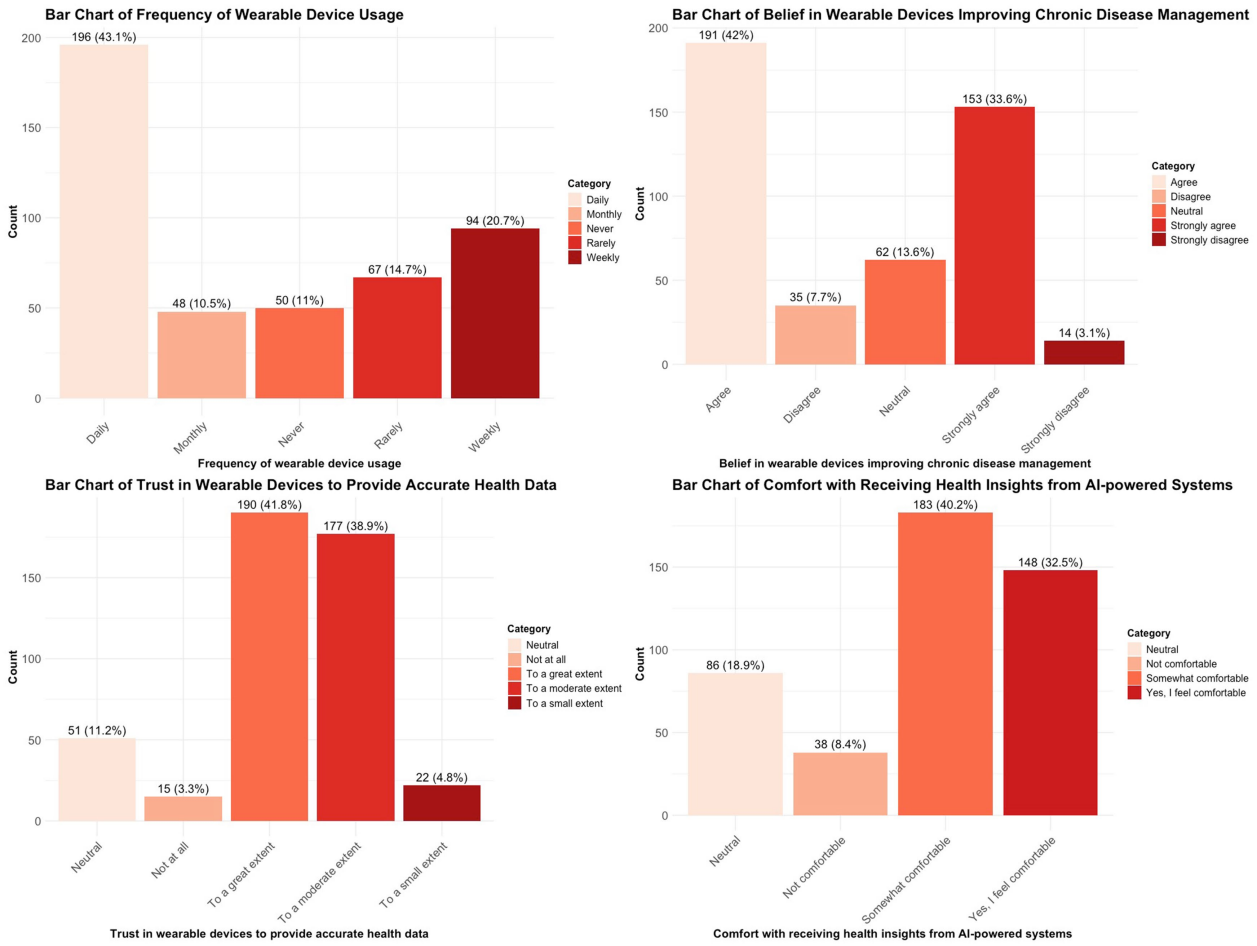
Frequency of trust and using of wearable devices and AI tools.

The bar charts in Figure 2 depict participants’ responses concerning their utilisation, trust, and perceptions of wearable devices and AI-driven systems for chronic disease management.

The data indicates that a majority of participants (194) reported using wearable devices on a “daily” basis, reflecting significant regular engagement with these technologies. A minority of participants indicated using wearable devices on a “weekly” (62), “monthly” (9), or “rarely” (17) basis, implying that daily users represent the predominant portion of the sample.

The belief in wearable devices’ capacity to enhance chronic disease management was largely affirmative, with the majority of respondents indicating “agree” (230) and “strongly agree” (143), thereby supporting the view of these devices as advantageous for proactive health monitoring.

Participants exhibited a strong level of trust in wearable devices for delivering accurate health data, with the majority responding “agree” (177) or “strongly agree” (159). However, a subset expressed “neutral” (89) or “disagree” (17) opinions, highlighting potential areas for reinforcing trust.

In terms of comfort with receiving health insights from AI-powered systems, the majority of participants expressed comfort, with 208 agreeing and 159 strongly agreeing. However, a smaller group of 88 remained neutral, indicating a degree of uncertainty that underscores the necessity of user education and trust-building for AI-based systems. The results indicate generally favourable perceptions; however, they highlight specific concerns regarding trust and comfort that should be addressed to improve adoption.

**Figure 3:**
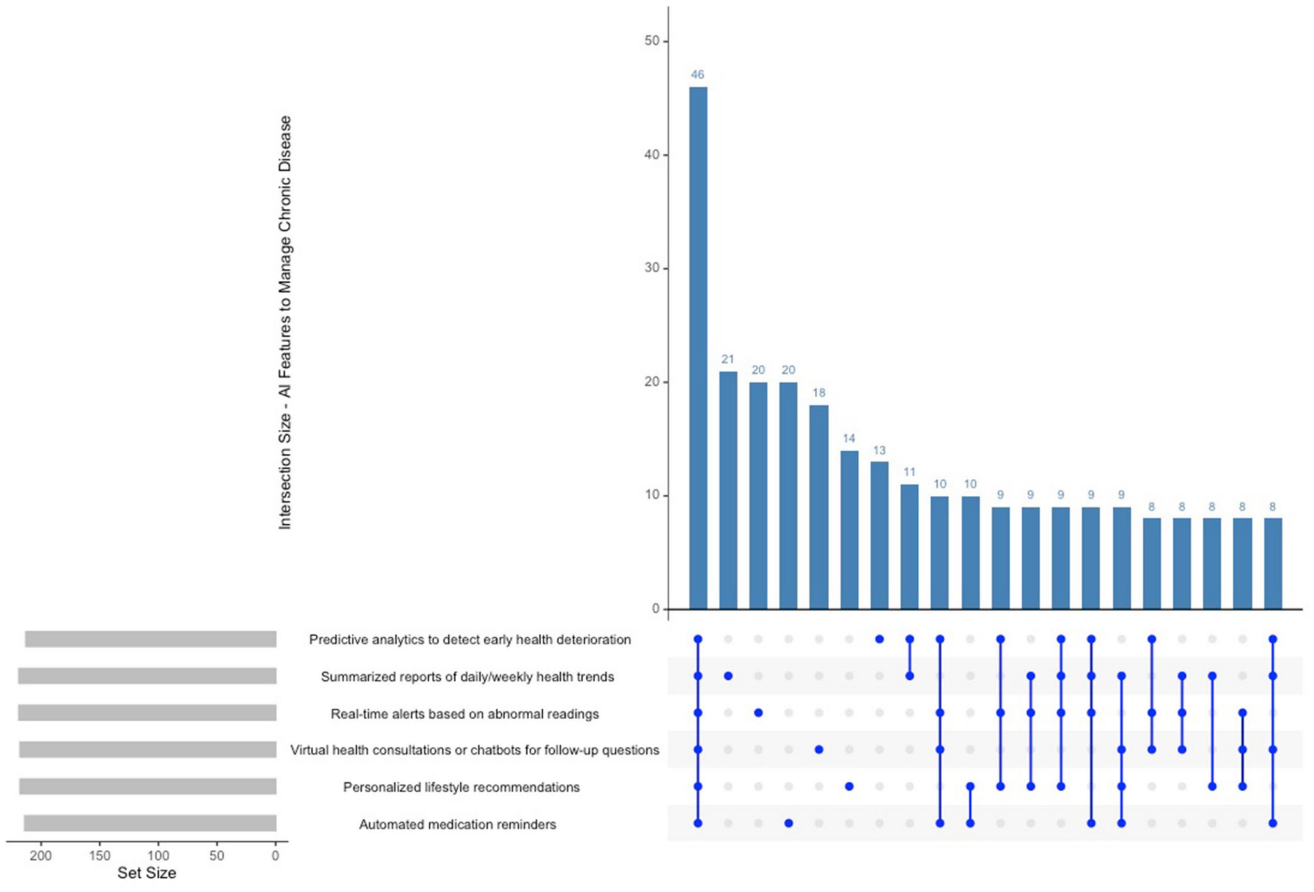
Upset plot AI features to manage chronic disease.

The UpSet plot in Figure 3 indicates that participants place significant importance on AI-driven features for chronic disease management, particularly highlighting predictive analytics for early health deterioration detection and real-time alerts for abnormal readings, both of which received 219 selections each. Furthermore, virtual health consultations and chatbots for follow-up enquiries were frequently selected, totalling 218 selections. The intersections indicate that numerous participants opted for multiple features simultaneously, reflecting a preference for integrated support that encompasses alerts, predictive insights, and virtual assistance. The predominant combination involved predictive analytics, real-time alerts, and virtual consultations, whereas automated health summaries and interactive feedback tools were less frequently chosen in conjunction.

**Figure 4:**
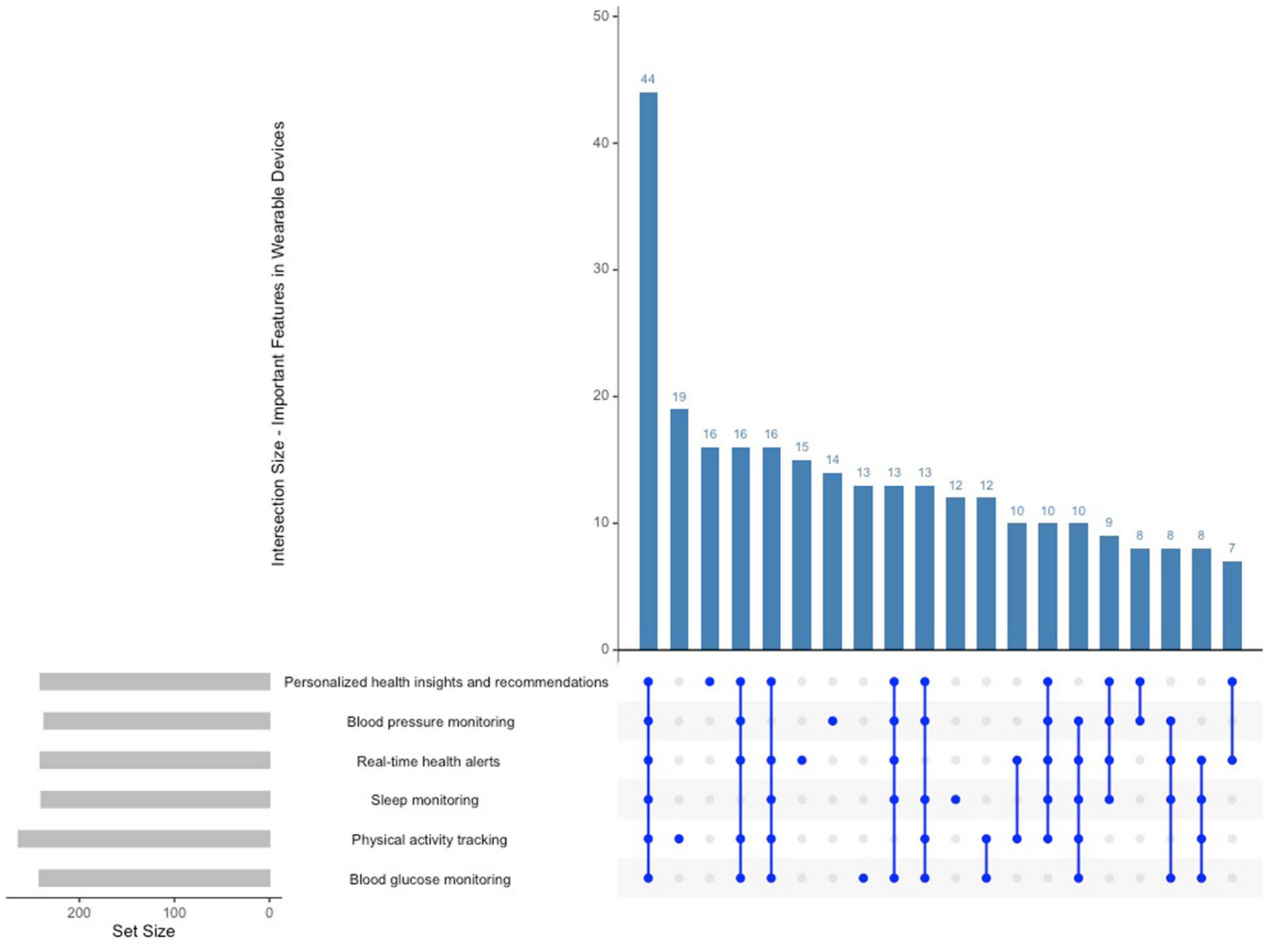
Upset plot important features in wearable devices.

As per Figure 4 participants ranked physical activity tracking (264 selections), blood glucose monitoring (242 selections), and sleep monitoring (241 selections) as the most significant features in wearable devices. Participants frequently exhibit a preference for wearables capable of simultaneously tracking multiple health metrics, including activity, sleep, and blood pressure. This indicates that users prioritise multi-functional devices that can deliver comprehensive health data, highlighting a demand for wearables that facilitate both lifestyle monitoring and chronic disease management. The most common combination involved physical activity, blood glucose, and sleep tracking, whereas temperature monitoring and oxygen saturation were less frequently chosen in conjunction.

**Figure 5:**
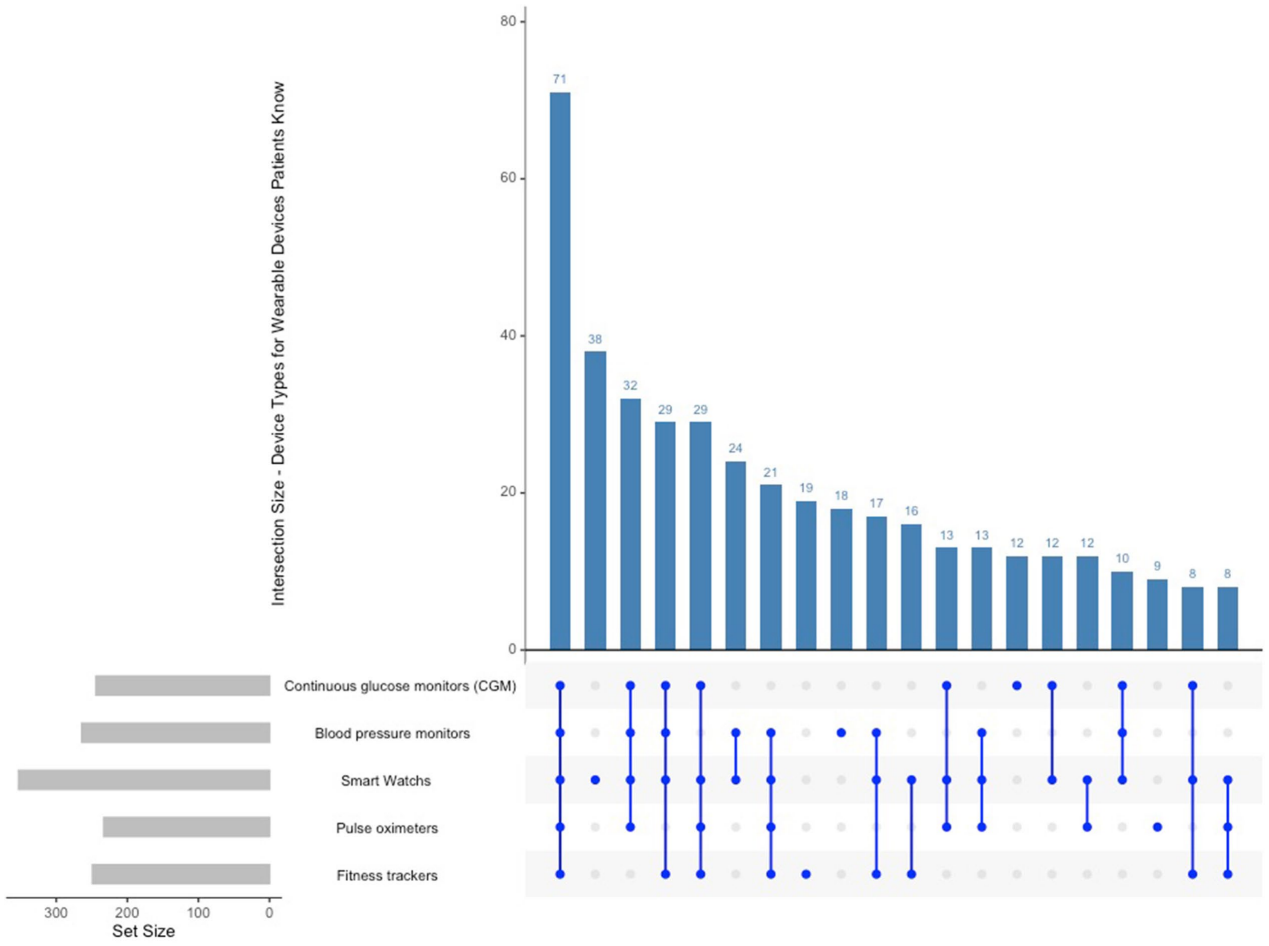
Upset plot types of wearable devices known to patients.

Among participants, the most recognised wearable devices were smartwatches, with 353 selections, followed by blood pressure monitors at 264 and fitness trackers at 249. Continuous glucose monitors (244) were widely recognised, indicating familiarity with devices typically linked to chronic disease management (see Figure 5). The prevalent familiarity with consumer-grade devices, including smartwatches and fitness trackers, indicates that patients are at ease with mainstream wearables; however, they may need additional education regarding medical-grade devices to maximise their health monitoring capabilities. The predominant intersection involved smartwatches, fitness trackers, and blood pressure monitors, whereas pulse oximeters and biosensor patches were less commonly recognised in conjunction.

**Figure 6:**
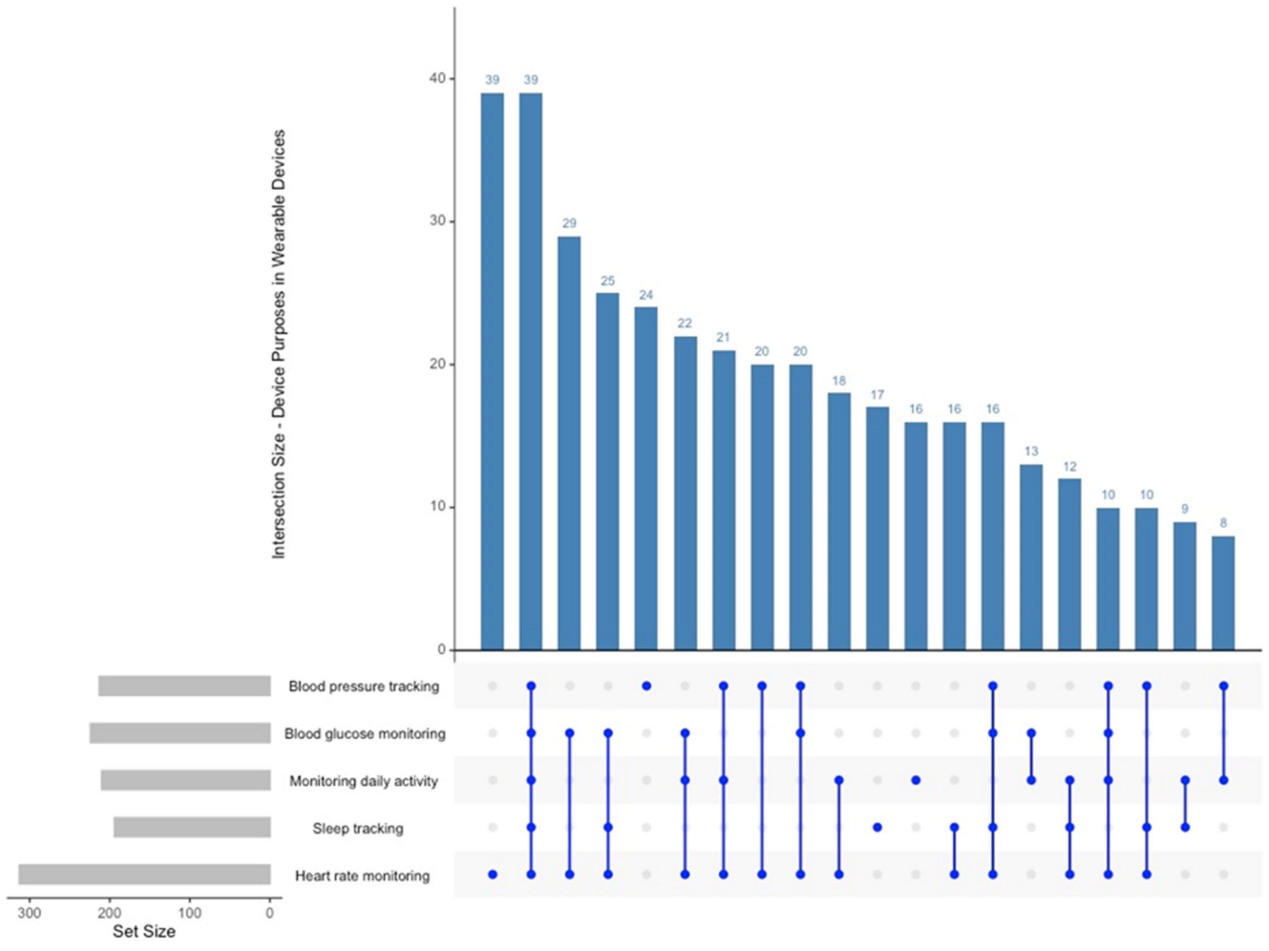
Upset plot device purposes in wearable devices.

Wearable devices are predominantly utilised by participants for heart rate monitoring (313 instances), daily activity tracking (224 instances), and sleep monitoring (210 instances) (see Figure 6). Blood glucose monitoring was frequently selected, underscoring the significance of vital sign tracking in the management of chronic diseases. The findings indicate that numerous participants utilise wearable devices for various functions, suggesting an expectation for wearables to facilitate extensive health monitoring rather than merely tracking singular activities. The predominant combination observed was heart rate, sleep, and daily activity monitoring, whereas temperature tracking and respiratory rate monitoring were less frequently chosen in conjunction.

As seen in Table 1 The findings indicate positive patient perceptions of wearable devices that incorporate artificial intelligence (AI) in healthcare, especially regarding chronic disease management and remote monitoring. A substantial number of participants concurred that these technologies enhance personalised care and enable real-time alerts to avert complications. For instance, 65.72% of respondents either agreed or strongly agreed that AI-powered tools can deliver reliable early diagnosis of complications related to chronic diseases (mean = 3.69). In a similar vein, 68.57% indicated confidence in the ability of AI-based tools to enhance the accuracy of disease monitoring (mean = 3.67), demonstrating patients’ trust in the potential of AI-driven diagnostics. Variability was noted, with 18.9 to 23.74% of respondents expressing neutrality on key items, reflecting cautious attitudes towards AI-based monitoring.

**Table 1:** Participant responses to statements regarding the perceived benefits, limitations, and applications of AI-integrated wearable devices for chronic disease management, expressed as percentages across Likert-scale categories.

Items	Strongly disagree (1)	Disagree (2)	Neutral (3)	Agree (4)	Strongly agree (5)	Mean score
Wearable devices integrated with AI can improve personalised care for chronic disease patients.	29 (6.37%)	43 (9.45%)	108 (23.74%)	173 (38.02%)	102 (22.42%)	3.61
AI chatbots that interpret wearable data can reduce the workload of physicians.	35 (7.69%)	33 (7.25%)	90 (19.78%)	183 (40.22%)	114 (25.05%)	3.68
Real-time alerts generated by AI from wearable device data can help prevent disease-related complications.	16 (3.52%)	41 (9.01%)	99 (21.76%)	190 (41.76%)	109 (23.96%)	3.74
I would trust an AI system to provide recommendations based on my wearable health data.	20 (4.4%)	49 (10.77%)	82 (18.02%)	183 (40.22%)	121 (26.59%)	3.74
Integration of wearable device data with AI can make remote consultations more effective.	16 (3.52%)	46 (10.11%)	81 (17.8%)	177 (38.9%)	135 (29.67%)	3.81
AI-powered tools can make sense of large volumes of wearable device data better than human healthcare providers.	25 (5.49%)	37 (8.13%)	95 (20.88%)	187 (41.1%)	111 (24.4%)	3.71
I am concerned that reliance on AI for health decisions could lead to a loss of human touch in patient care.	21 (4.62%)	46 (10.11%)	94 (20.66%)	173 (38.02%)	121 (26.59%)	3.72
AI-driven interpretations of wearable device data need to be supervised by healthcare professionals to ensure accuracy.	24 (5.27%)	55 (12.09%)	96 (21.1%)	168 (36.92%)	112 (24.62%)	3.64
Wearable devices integrated with AI can provide reliable early diagnosis of chronic disease complications.	26 (5.71%)	49 (10.77%)	86 (18.9%)	171 (37.58%)	123 (27.03%)	3.69
I believe wearable devices, when combined with AI, can help in more accurate monitoring of disease progression.	24 (5.27%)	45 (9.89%)	102 (22.42%)	170 (37.36%)	114 (25.05%)	3.67
AI-based tools using wearable device data are safe for chronic disease management without frequent in-person consultations.	20 (4.4%)	43 (9.45%)	91 (20.0%)	180 (39.56%)	121 (26.59%)	3.75
Wearable devices with AI can increase patient adherence to chronic disease management plans by providing personalised feedback and reminders.	22 (4.84%)	34 (7.47%)	93 (20.44%)	186 (40.88%)	120 (26.37%)	3.76
AI-driven diagnostics based on wearable device data can potentially reduce diagnostic errors for chronic diseases.	37 (8.13%)	41 (9.01%)	104 (22.86%)	165 (36.26%)	108 (23.74%)	3.58
I believe wearable devices integrated with AI are an effective tool for promoting proactive healthcare rather than reactive healthcare.	16 (3.52%)	43 (9.45%)	94 (20.66%)	186 (40.88%)	116 (25.49%)	3.75
I feel confident that AI systems analysing wearable device data can maintain patient safety in chronic disease care.	28 (6.15%)	53 (11.65%)	79 (17.36%)	184 (40.44%)	111 (24.4%)	3.65
I am concerned that technical failures in wearable devices or AI systems could lead to inaccurate diagnoses or delays in treatment.	25 (5.49%)	39 (8.57%)	89 (19.56%)	189 (41.54%)	113 (24.84%)	3.72
Integration of wearable devices with AI-based systems can help reduce healthcare costs associated with chronic disease management.	19 (4.18%)	45 (9.89%)	97 (21.32%)	188 (41.32%)	106 (23.3%)	3.7
I believe wearable devices with AI-based support can increase patient engagement in their own care.	23 (5.05%)	51 (11.21%)	85 (18.68%)	179 (39.34%)	117 (25.71%)	3.69

Patient feedback indicated the perceived influence of AI tools on healthcare efficiency. 68.57% of participants indicated agreement that AI-powered wearable devices could diminish the necessity for frequent in-person consultations (mean = 3.75), reflecting substantial endorsement for remote consultations. Additionally, 67.25% concurred that these tools have the potential to alleviate physician workload through effective data interpretation (mean = 3.68). Concerns regarding technical failures and their potential impact were significant, with 66.38% of respondents agreeing that device malfunctions could result in inaccurate diagnoses or treatment delays (mean = 3.72). The concerns highlight the necessity of dependable technology and technical support to ensure patient safety.

The results underscore the significance of AI in facilitating proactive care. 66.37% of participants indicated that AI-powered wearable devices have the potential to transition healthcare from a reactive to a proactive model (mean = 3.75). Additionally, 67.25% concurred that personalised feedback and reminders may improve adherence to chronic disease management plans (mean = 3.76). Nonetheless, the necessity for professional oversight was apparent, with 61.54% concurring that AI-generated analyses of wearable data require monitoring by healthcare professionals (mean = 3.64). The findings indicate that patients perceive AI-integrated wearable devices as beneficial for enhancing healthcare outcomes. However, it is crucial to address concerns regarding accuracy, safety, and technical reliability to facilitate wider adoption and maintain trust.

The findings in Table 2 show no statistically significant differences in perceptions of AI-integrated wearable devices when analysed across demographic and belief-based variables. The Kruskal-Wallis test indicated negligible differences concerning familiarity with wearable devices (*p* = 0.87), device usage (*p* = 0.59), and department (*p* = 0.45). The Mann–Whitney U test indicated no significant differences related to chronic condition status (*p* = 0.35) or gender (*p* = 0.65). Perceptions were uniform across groups, reflecting a consensus on the advantages and difficulties associated with wearable health technologies.

**Table 2:** Results of Kruskal-Wallis and Mann–Whitney U tests assessing differences in composite scores for perceptions of AI-integrated wearable devices across demographic and belief-based variables.

Independent variable	Kruskal-Wallis statistic	*p*-value	Mann–Whitney U statistic
Familiarity with wearable devices	0.29	0.87	
Wearable devices usage	1.92	0.59	
Belief in wearables for disease management	3.87	0.14	
Trust in wearables’ accuracy	1.69	0.43	
Comfort with ai-powered insights	0.38	0.83	
Department	2.63	0.45	
Chronic condition		0.35	5979.00
Gender		0.65	5758.00

## Discussion

This study explored patients’ perceptions, trust, and awareness of wearable devices incorporating artificial intelligence (AI) for chronic disease management. The findings predominantly indicated a positive attitude towards AI-integrated wearable devices, particularly in their ability to enhance personalised care, provide real-time health insights, and facilitate proactive health monitoring. These findings align with previous research, which has consistently highlighted the transformative potential of wearable health technologies in chronic disease management through real-time monitoring and personalised interventions (1, 5). Research has demonstrated that these devices enable real-time predictive health monitoring, improving early detection and personalised treatment approaches for chronic conditions such as diabetes and cardiovascular diseases (23).

Participants exhibited significant trust in these devices, with most agreeing that wearable technologies improve the accuracy of disease monitoring and provide reliable early diagnoses. This trust is in line with previous studies that have found patients perceive AI applications as beneficial, particularly in enhancing diagnostic precision and treatment efficiency (3, 10). The issue of trust remains pivotal in the adoption of AI-integrated wearables. Existing research has shown that trust is heavily influenced by factors such as data security, transparency in AI decision-making, and the integration of these technologies into clinical workflows. Joshi (24) asserts that AI should be regarded as a complementary tool rather than a replacement for traditional healthcare, reinforcing trust through a hybrid model that combines AI assistance with physician supervision (24). Similarly, Patel et al. (25) underscore that trust in remote AI-driven monitoring is closely tied to robust data privacy measures and the mitigation of algorithmic bias.

Despite the overall positive reception, concerns remain regarding data accuracy, technical failures, and the potential reduction of human interaction in healthcare. These concerns echo findings from previous studies that have identified data quality and the erosion of the doctor-patient relationship as significant barriers to AI adoption in clinical practice (20). Notably, between 18.9 and 23.74% of respondents expressed neutrality on certain aspects of AI-driven recommendations, suggesting reservations about their reliability. This caution is consistent with previous studies in which participants expressed uncertainty regarding the safety and interpretability of automated health insights (3, 17). Furthermore, while AI-powered tools such as virtual health assistants received substantial support, a majority of respondents emphasised the necessity of healthcare provider oversight to ensure accuracy, reinforcing the importance of human involvement in AI-assisted healthcare.

While AI-driven wearable technology is generally well-received, challenges persist regarding usability and the potential overreliance on automation. Concerns about algorithmic bias have been substantiated by studies demonstrating disparities in AI-based health predictions across different demographic groups. AI models trained on non-representative datasets may produce skewed health insights, resulting in biased predictions and unintended disparities in health outcomes (26). Addressing these issues requires continuous refinement of AI algorithms, ensuring the inclusion of diverse and representative datasets to promote equitable health outcomes. Wang, Asan (27) stress that AI-based homecare systems must be implemented with appropriate oversight to optimise chronic disease management without compromising patient safety (27).

This study found no statistically significant differences in perceptions based on demographic factors such as age, gender, or chronic disease status, suggesting that AI-integrated wearable devices have the potential for widespread acceptance across diverse populations, provided that existing concerns are adequately addressed. However, individuals with chronic conditions exhibited more favourable attitudes towards the utility of these devices, aligning with existing literature indicating that patients with chronic diseases are more likely to adopt wearable technologies for continuous health monitoring (4, 14).

Beyond usability and privacy concerns, AI-integrated wearable technologies pose additional challenges, particularly with algorithmic bias and the risk of excessive reliance on automation. AI models embedded in wearable devices rely on existing datasets, which may not always be representative of diverse patient populations, potentially leading to biased predictions and inaccurate health recommendations. Such biases have been widely documented, particularly in relation to racial, gender, and socioeconomic disparities in AI-driven health assessments (6, 21). Addressing these issues necessitates the ongoing refinement of AI algorithms through the incorporation of diverse and representative datasets, as well as transparent model validation processes.

Furthermore, while AI automation enhances efficiency, there is a danger that both patients and healthcare providers may become overly reliant on AI-generated insights without applying sufficient critical evaluation. Such dependence may lead to delayed or incorrect diagnoses if AI predictions are accepted uncritically, highlighting the need for AI to function as an adjunct rather than as a sole decision-maker in healthcare. To mitigate this risk, patient education initiatives should emphasise AI’s role as a decision-support tool rather than as a definitive authority. Additionally, integrating AI-generated recommendations within established clinical oversight protocols can ensure a balanced approach that combines algorithmic insights with human expertise.

Accessibility remains a critical issue, particularly for individuals from lower socioeconomic backgrounds. Research has highlighted that financial constraints and limited digital literacy often hinder the widespread adoption of AI-integrated wearable technologies. Rath, Khang (28) propose that the development of affordable and user-friendly AI-powered devices, coupled with targeted educational interventions, could enhance accessibility and usability for diverse patient populations (28). Addressing these challenges through intuitive user interface designs, cost-effective device options, and tailored educational initiatives could significantly improve the adoption and effectiveness of AI-powered wearable technologies.

To enhance the adoption of AI-integrated wearable devices, policymakers should prioritise digital health literacy initiatives, ensuring patients, particularly those with lower digital proficiency, can effectively engage with these technologies. Integrating AI-driven wearables into public healthcare systems or providing financial subsidies would improve accessibility, especially for those facing socioeconomic barriers. Clinicians play a vital role in bridging the trust gap by offering clear guidance on AI-generated recommendations and ensuring these technologies are seen as complementary rather than substitutive to human expertise. Healthcare institutions should implement structured training programs to equip clinicians with the skills to interpret AI-driven insights and communicate them effectively to patients. Additionally, regulatory bodies must establish transparent AI governance frameworks and enforce algorithmic fairness to ensure equitable and unbiased health outcomes. Addressing these challenges will support the responsible and widespread integration of AI-powered wearables, ultimately improving patient engagement and health outcomes in chronic disease management.

While this study acknowledges concerns regarding data privacy, security, and algorithmic bias in AI-integrated healthcare technologies, it does not provide a comprehensive analysis of the ethical and legal frameworks that govern their use. Given the increasing reliance on AI-driven health monitoring, future research should critically examine regulatory frameworks such as the General Data Protection Regulation (GDPR) and the Health Insurance Portability and Accountability Act (HIPAA). These regulations establish essential safeguards for data protection, patient consent, and security, yet their applicability to AI-driven health technologies remains an evolving challenge, particularly regarding cross-border data sharing, informed consent in automated decision-making, and liability for algorithmic errors. A more detailed evaluation of how these regulations address emerging ethical dilemmas is necessary to ensure AI-integrated wearables operate within a transparent and accountable legal framework.

Beyond regulatory compliance, algorithmic bias poses a significant risk in AI-driven health analytics, potentially leading to disparities in diagnostic accuracy and treatment recommendations. AI models trained on historically skewed datasets may not adequately represent diverse patient populations, exacerbating health inequalities. Future research should focus on bias mitigation strategies, including greater diversity in training datasets, ongoing algorithmic audits, and regulatory oversight to ensure fairness. Moreover, increasing the explainability of AI-generated insights will be crucial in fostering trust among both patients and healthcare providers. Interdisciplinary collaboration between technologists, ethicists, and clinicians, alongside targeted patient education initiatives, will be essential in ensuring that AI-driven healthcare remains equitable, transparent, and centred on patient well-being.

### Strengths, limitations, and recommendations for future work

This study provides a comprehensive analysis of patients’ perceptions, trust, and awareness regarding AI-integrated wearable devices for chronic disease management. A key strength lies in its demographic inclusivity, offering insights into how different patient groups engage with AI-powered health technologies. The inclusion of multiple-choice questions assessing awareness of AI-driven features enhances the findings by measuring participants’ familiarity with predictive analytics, automated symptom monitoring, and AI-generated health recommendations. Furthermore, the use of validated survey instruments and pilot testing ensured clarity and reliability, improving the internal validity of the study.

However, several limitations must be acknowledged. The use of convenience sampling may have introduced selection bias, potentially limiting the generalisability of findings. Future research should employ randomised sampling to enhance external validity. Additionally, the reliance on self-reported data presents risks of recall bias and social desirability effects, where participants may have overestimated their familiarity with AI-integrated wearables or responded in ways they perceived as desirable. To mitigate these issues, future studies should integrate objective behavioural data from wearable devices to provide a more accurate measure of user engagement and trust. Furthermore, the lack of longitudinal follow-up prevents assessment of how perceptions evolve over time, highlighting the need for long-term studies tracking changes in trust, usability, and adherence to AI-powered healthcare solutions.

This study was conducted within a specific geographical context (Saudi Arabia), which may limit the generalisability of findings to other cultural and economic settings where AI adoption barriers and healthcare infrastructures differ. Future research may need to explore cross-cultural comparisons to assess how sociocultural, economic, and regulatory factors influence perceptions of AI-driven wearable devices. Additionally, while concerns about AI bias and data security were acknowledged, the study did not examine how these issues vary across demographic groups. Given disparities in digital literacy and access to health technology, future research should investigate how socioeconomic status and health literacy shape trust in AI-integrated healthcare. Addressing these concerns through inclusive AI design, education initiatives, and transparent validation processes will be critical to fostering equitable access and patient confidence in wearable health technologies.

## Conclusion

This study highlights patients’ favourable views of AI-integrated wearable devices in healthcare, especially regarding personalised care and remote monitoring support. The results demonstrate widespread acceptance among various demographic groups, with patients appreciating features like real-time alerts, predictive insights, and virtual health assistance. Key concerns regarding technical reliability, data accuracy, and the diminished human interaction in care were identified as obstacles to broader adoption. The research highlights the necessity of professional supervision to uphold trust and guarantee precision in AI-driven recommendations. Targeted education, simplified interfaces, and enhanced support systems can effectively address barriers, thereby improving the usability and adoption of wearable health technologies. Future research should prioritise longitudinal studies to evaluate the long-term effects of AI-driven wearables and develop strategies to address patient concerns while advancing proactive, technology-enabled healthcare.

## Data Availability

The raw data supporting the conclusions of this article will be made available by the authors, without undue reservation.
